# Tracheal Intubation through the I-gel for Emergency Cesarean Section in a Patient with Multidrug Hypersensitivity: A New Technique

**DOI:** 10.1155/2014/245752

**Published:** 2014-07-20

**Authors:** Kartika Balaji Samala, Yuri Uchiyama, Yasuyuki Tokinaga, Yukitoshi Niiyama, Soshi Iwasaki, Michiaki Yamakage

**Affiliations:** ^1^Sapporo Medical University School of Medicine, Sapporo, S1W16,Chuo-ku, Sapporo-shi, Hokkaido 060-8543, Japan; ^2^GSL Medical College and Hospital, Rajahmundry, India

## Abstract

31-year-old female with hypersensitivity to local anesthetics and neuromuscular blocking agents presented for emergency Cesarean section. We successfully performed I-gel-assisted tracheal intubation without using neuromuscular blockers. We believe this method would be helpful in selected situations.

## 1. Introduction

Allergic reactions to anesthetics, drugs, blood products, and neuromuscular blocking agents (NMBA) have been reported during anesthesia [1]. These are sometimes life threatening and difficult to deal with. Our case report highlights the successful management of a patient who presented for Cesarean section with allergy to both NMBAs and local anesthetics on skin testing. Anesthesia was induced with propofol, fentanyl, and the inhalational agent, sevoflurane. A Parker tracheal tube inserted through an I-gel under fiberoptic bronchoscopy was used to secure the airway. We received permission from the patient and restored in electronic medical record to publish this report.

## 2. Case Report

**Figure 1:**
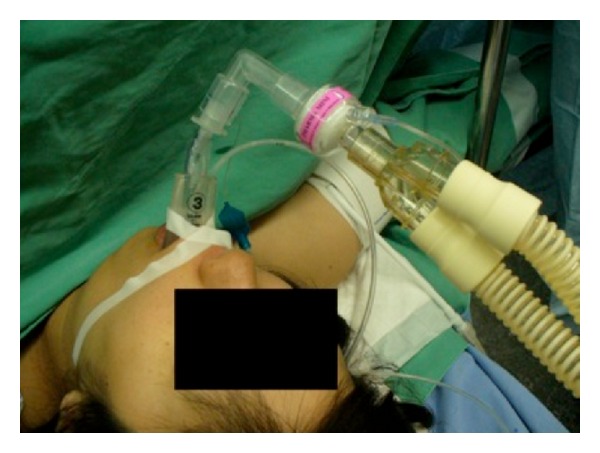
Insertion of the Parker tracheal tube and a nasogastric tube through the I-gel.

A 31-year-old female, 158 cm tall and weighing 73.8 kg who had regular antenatal visits, came for the safe institutional delivery. Her medical history dated back to 5 years, with a history of Steven-Johnson syndrome and allergy to carbamazepine. She reported a past history of allergy to lidocaine, procaine, bupivacaine, chlorpheniramine maleate, diclofenac sodium, serratiopeptidase, latex, raw eggs, crabs, and iodine. She had chronic adrenal insufficiency for which she was treated with steroids, which, however, resulted in osteoporosis. Anesthesiologists, obstetricians, and dermatologists discussed the patient's condition and decided to manage her under general anesthesia if normal vaginal delivery was not possible. As per the guidelines for conduct of general anesthesia [2] in such patients, intradermal skin tests for drug allergies were performed, which were positive for lidocaine, procaine, bupivacaine, suxamethonium, and rocuronium at 1 : 100 dilutions as previously reported [3]. The patient was given a trial of normal vaginal delivery in the labor room. However, emergency Cesarean section was required due to nonprogression of labor and fetal distress. Once she arrived in the operating room, oxygen was delivered via a face mask. Anesthesia was induced with 140 mg of propofol, N_2_O : O_2_ in a ratio of 4 : 2 l/min, and sevoflurane at an end-tidal concentration of 2%. A supraglottic device, the I-gel size 3, was used to secure the airway, seal pressure being maintained above 20 cm H_2_O before the start of surgery. When surgery was commenced, the patient did not show any motor activity, indicating an adequate depth of anesthesia. At the time of the uterine incision, a 6.5 mm Parker tracheal tube (Parker Flex-tip, Colorado, USA) was inserted through the I-gel under fiberoptic bronchoscopy without any resistance. Six ml of air was used to inflate the cuff and bilateral air entry was confirmed by auscultation of the chest. The baby was successfully delivered and had APGAR scores of 7 and 9 at 1 and 5 min, respectively. The mother was given 200 *μ*g of fentanyl immediately after giving birth of the baby. Suctioning using a 10 Fr catheter (Createmedic, Yokohama, Japan) through the I-gel revealed the presence of 8 mL of gastric juice (Figure 1). Postoperatively, the patient was extubated in the recovery room after emergence from anesthesia. Once she was moved to the ward, she complained of mild itching over the forearm, which was not considered to be significant as she was hemodynamically stable. Postoperatively, both mother and baby did well and were discharged from the hospital after a few days without any complications.

## 3. Discussion

Anesthesia-related maternal mortality during Cesarean sections was reported by Hawkins et al. between 1980–90 and 1997–2002. The mortality ratios of general anesthesia to local anesthesia were about 9.8 times [4]. Since local anesthesia very rarely causes severe complications resulting in death, it is preferred over general anesthesia for most surgeries, unless there is an absolute contraindication to its use. General anesthesia is currently adopted when there is no time or when regional anesthesia fails, and it is limited to patients who refuse regional anesthesia or have an abnormal coagulation profile, spinal cord disorders, and allergic reactions to local anesthetics, as in our case.

In our patient, intradermal allergy tests revealed allergy to both ester and amide local anesthetics, limiting their usage. Hence, we selected general anesthesia using drugs that were considered safe in this patient.

Conduct of general anesthesia in parturients can be by (1) rapid sequence induction, (2) awake intubation, or (3) volatile induction. The gold standard for Cesarean section during general anesthesia is rapid sequence induction using thiopentone as the induction agent and suxamethonium as the depolarizing agent to facilitate intubation [5]. Currently, propofol is often used as the induction agent, with rocuronium as the NMBA.

Positive intradermal allergy tests to both depolarizing and nondepolarizing NMBAs in our patient limited their usage, as they are the largest cause of anaphylaxis after induction of anesthesia [2]. We ruled out rapid sequence induction in our patient, as NMBAs could not be used. We also ruled out awake intubation with remifentanil, as the methods would be too slow. Though anaphylaxis cannot be expected in every patient with a positive intradermal test, considering the safety of the patient and her past history of drug allergies, we chose to conduct anesthesia in the manner described here.

Laryngospasm and the risk of aspiration were our main concerns during the conduct of anesthesia in this patient. The sympathetic response to surgery may cause laryngospasm if the depth of anesthesia is inadequate. Use of topical anesthetics to blunt the sympathetic response had to be limited in our patient due to the risk of anaphylaxis. Insertion of the tracheal tube through the I-gel helped overcome this problem, while still maintaining a short time interval between I-gel insertion and intubation. The patient also remained hemodynamically stable during this period. Volatile induction was not considered feasible in our patient since it was an emergency Cesarean section. Our technique enabled avoidance of NMBA usage and showed that, in some difficult airway scenarios during emergency Cesarean section, our technique is easy and saves time.

Opioids are mainly used during general anesthesia to obtund the neuroendocrine stress response and for hemodynamic stability. However, it is not clear how quickly fentanyl crosses the placenta. Hence, keeping in mind the risk of fetal respiratory depression and low APGAR scores, its use was precluded in our patient before delivery of the baby [6]. Halaseh et al. [7] reported that use of the Proseal laryngeal mask airway was effective in protecting against the risk of aspiration in 3000 elective Cesarean section patients with a minimum fasting period of 8 hr. In our patient, we opted to use the I-gel as it has the advantage of more rapid and easier insertion and decreased incidence of gastric insufflation when compared to laryngeal mask airways (LMA), both of which were desirable in our patient. Very few studies or case reports have described use of the I-gel and its advantages over the classic LMA, except for one previous study that supports our work [8]. These reports suggest the utility of supraglottic airway device* per se* in cesarean section. The 2nd generation supraglottic airway device such as proseal LMA(PLMA), intubating LMA(ILMA) could be alternatives in our case. Kleine-Brueggeney reported the success of fibreoptic intubation which was similar between using I-gel and ILMA, and the former was superior to ILMA in terms of time to insertion [9]. Air Q is one supraglottic airway device which could pass tracheal tube as easy as I-gel in the manikin study [10]; however it was necessary to take off the tip which wasted crucial time in scenarios like our patient. So, we choose to use I-gel as a supraglottic device considering our patient's condition. Here, we emphasize that tracheal intubation through the I-gel is a new method in particular for Caesarian section which helps in the progress of anesthesia and surgery at the same time.

Our case report highlights a novel approach of I-gel-assisted tracheal intubation in patients in whom NMBAs cannot be used.
